# Editorial: Relationship between gestational and neonatal diabetes mellitus

**DOI:** 10.3389/fendo.2022.1060147

**Published:** 2022-10-14

**Authors:** Ihtisham Bukhari, Furhan Iqbal, Rick Francis Thorne

**Affiliations:** ^1^ Translational Research Institute, Henan Provincial and Zhengzhou City Key Laboratory of Non-coding RNA and Cancer Metabolism, Henan International Joint Laboratory of Non-coding RNA and Metabolism in Cancer, Henan Provincial People’s Hospital, Academy of Medical Sciences, Zhengzhou University, Zhengzhou, China; ^2^ Henan Key Laboratory of Helicobacter pylori, Microbiota and Gastrointestinal Cancer, Marshall Medical Research Center, Fifth Affiliated Hospital of Zhengzhou University, Zhengzhou, China; ^3^ Institute of Zoology, Bahauddin Zakariya University, Multan, Pakistan; ^4^ School of Environmental and Life Sciences, University of Newcastle, Callaghan, NSW, Australia

**Keywords:** Gestational Diabetes – Mellitus, Neonatal Diabetes Mellitus, Metabolic Disorder, Metabolic Syndrome, Pregnancy Complications

Diabetes is a global concern that increases the risk for coronary heart disease, stroke, and other pathological conditions. Notably, a relationship exists between maternal metabolic syndrome (MetS), gestational diabetes mellitus (GDM), and pregnancy outcomes (1). GDM manifests as glucose intolerance during gestational weeks 24-28 (2) and may cause short or long-term health complications for the mother, fetus, or both (3) including maternal psychological disturbance, severe birth trauma due to fetal overgrowth, fetal death and stillbirth (4). Compared to the common forms of diabetes, the causes and associated complications of GDM and neonatal diabetes mellitus (NDM) are not well understood. Moreover, the scientific consensus is lacking regarding the proper diagnosis of GDM and its unreported effects on the fetus (5). Recently, we published a series of reports describing various causes, comorbidities and diagnostic approaches for GDM and NDM (6). The current research topic aimed to collect studies reporting advancements in clinical and basic research related to GDM, NDM, and associated metabolic disorders. After a rigorous selection and review process, the current volume presents an authoritative collection of seven research articles exploring new dimensions of this topic.

The first article from this collection explores the topic of diagnostic approaches for GDM. Beunen et al. used type 1 diabetes (T1D)-related autoimmune antibodies to characterize women suffering from GDM and their long-term risk of glucose intolerance. They found a low sensitivity rate of the T1D-related autoantibodies in pregnant and postpartum women concluding that the use of T1D-related autoantibodies for GDM diagnosis was unwarranted.

A retrospective analysis of perinatal outcomes of twin GDM pregnancies was performed by Lin et al.. The authors compared data from GDM and non-GDM Chinese women and interestingly, reported a GDM incidence of 21.9%. The women with GDM were older, overweight, and/or obese and were associated with chronic hypertension, assisted pregnancies, and dichorionic twins. Notwithstanding these findings, no significant differences were found between perinatal outcomes among GDM and non-GDM women. The authors concluded that aging and metabolic health play a critical role in GDM and associated maternal and fetal complications.

Due to the increasing rate of diabetes, exploring GDM-associated complications in mothers and neonates has become a hot research topic in recent years. Most studies have revolved around the causes and effects of diabetes (non-GDM, GDM, and NDM). In the same context, Kumar and Diamond reviewed the literature on pregnancy outcomes in GDM women fasting in Ramadan: the holy month in the Islamic calendar. The available literature found no significant correlation between fasting during Ramadan and pregnancy outcomes in GDM women. Based on the effects of the long hours of fasting, the authors recommended no fasting for both GDM and non-GDM women during Ramadan. However, the editors’ opinion on this matter is that insufficient literature support is currently available regarding pregnancy outcomes in fasting GDM and Non-GDM women; thus, large-scale multicentered studies should be performed to get a more accurate picture.

Tang et al. performed a systematic review and meta-analysis regarding the efficacy and safety of Evogliptin for type 2 diabetes mellitus. They found similarities in the effectiveness of Evogliptin compared to other DPP-4i drugs, including Sitagliptin and Linagliptin, for managing HbA1c levels and adverse events associated with the disease. Additionally, Jiang et al. studied the association between diabetic foot ulcer (DFU) with dysmetabolism and other factors in diabetic patients. They used 12 risk factors to construct the nomogram, which reliably predicted the risk of DFU in patients with T2D. As it is already a fact that diabetes or stimulation of blood glucose causes several diseases such as DFU and degenerative brain disease (DBD), Liu et al. studied the underlying mechanisms of Gardin (an actin-binding protein) in DBD development resulting from glucose stimulation. They observed that upon glucose stimulation, Gardin and related signaling pathways such as Akt and STAT3 significantly decrease in neurons leading to brain degeneration.

Finally, Agius et al. analyzed the differences in leukocyte mitochondrial copy numbers in individuals with metabolic syndrome (MetS) and metabolically healthy obese. A reduced leukocyte mitochondrial DNA (mtDNA) copy number was detected in MetS patients, and a significant association was observed between the reduced leukocyte mtDNA copy number, obesity and metabolic syndrome. These findings suggested a crucial role of mitochondria in the development of metabolic disorders such as Mets and diabetes, but underlying mechanisms are not entirely understood.

## Conclusions and perspectives

In the current research theme, seven research groups have highlighted different aspects of diabetes/gestational diabetes and metabolic disorders. These studies have added new information to the existing knowledge regarding the diagnosis of gestational diabetes, pregnancy outcomes in GDM women and other diabetes-associated phenotypes. Although T1D-related autoantibodies were detected in some four years of postpartum women, the results are not compelling due to less population size and low sensitivity of the autoantibodies. Thus, leaving a significant question of whether or not GDM triggers NDM or postnatal diabetes. We expect that these contributions will find broad applications, ranging from purely scientific endeavors to clinical guidelines for treating diabetic patients.

## Author contributions

All authors listed have made a substantial, direct, and intellectual contribution to the work and approved it for publication.

## Conflict of interest

The authors declare that the research was conducted in the absence of any commercial or financial relationships that could be construed as a potential conflict of interest.

## Publisher’s note

All claims expressed in this article are solely those of the authors and do not necessarily represent those of their affiliated organizations, or those of the publisher, the editors and the reviewers. Any product that may be evaluated in this article, or claim that may be made by its manufacturer, is not guaranteed or endorsed by the publisher.
